# Heat Transfer Performance of Gel Foam Layer with Nanoparticles Doping under a Radiative Heat Flux

**DOI:** 10.3390/mi13122223

**Published:** 2022-12-14

**Authors:** Rifeng Zhou, Pengyu Cui, Qingli Cheng, Xuqing Lang, Yong Zhang, Qie Sun, Mu Du

**Affiliations:** 1 State Key Laboratory of Safety and Control for Chemicals, SINOPEC Research Institute of Safety Engineering Co., Ltd., Qingdao 266000, China; 2 School of Energy Science and Engineering, Harbin Institute of Technology, Harbin 250001, China; 3 Institute for Advanced Technology, Shandong University, Jinan 250061, China; 4 MOE Key Laboratory of Thermo-Fluid Science and Engineering, Xi’an Jiaotong University, Xi’an 710049, China

**Keywords:** multi-phase, gel foam, insulation, nanoparticle

## Abstract

The risk of fire in the chemical industry’s production process is fatal. Gel foam has been widely employed in petroleum storage tanks, oil pools, and other petrochemical equipment for fire extinguishing and thermal protection. Recently, nanoparticles have been doped into gel foam to enhance thermal stability and insulation. However, heat transfer behaviors of the gel foam layer containing nanoparticles are still missing. In this study, a numerical heat transfer model of a gel foam layer containing silica nanoparticles under a radiative heat flux was established. Through simulation, the changes in foam thickness and temperature distribution were analyzed. The effects of the maximum heating temperature, initial gas content, nanoparticle size, and concentration on the thermal insulation behavior of the gel foam layer were systematically studied. The results showed that the thermal stability and insulation performance of the three-phase gel foam layer decreased with the increase in the initial gas content and particle size. Increasing the nanoparticle concentration could enhance the foam’s thermal stability and insulation performance. The results provide guidance for a designing gel foam with high thermal protection performance.

## 1. Introduction

Oil storage safety is of great significance to the economic development and national security. Abnormal risk factors such as the lightning strike can cause oil depot fires, which may result in huge economic losses and pollution to the surrounding environment [1]. When an unexpected fire occurs in an oil depot, the thermal radiation temperature of the black smoke from the oil fire is about 800 K [2], and the thermal radiation intensity may reach to 30 kW m^−2^ [2,3]. So it is easy to cause fire of the adjacent oil tanks, or even a big fire spread to the whole oil depot, if the fire protection or rescue is delayed. Therefore, measures must be taken to enhance the thermal protection of adjacent tanks in fire. Gel foam can be a solution. The gel foam is mainly composed of gas and gel phases. In the gel phase, water is the main component, and there is also a small amount of thickening and crosslinking agents. Water in the gel foam has a significant contribution to the thermal resistance of foam [4]. First, water has a large specific heat capacity, which increases the latent heat of gel foam. Second, water in the gel foam can absorb and diffuse the thermal radiation, which is beneficial to the radiation thermal resistance. Third, water in the gel foam has a cooling effect in the high temperature environment by evaporating. If the gel foam contains particles, the particles will also contribute to the thermal insulation. Gel foam containing particles can be termed as a three-phase gel foam.

The unit structure in foam consists of four adjacent bubbles. A thin film is sandwiched between two adjacent bubbles, so the thin film is also known as a bubble wall. A Plateau border with a concave triangular cross-section is also formed around every three adjacent bubbles. The gel phase with liquid in the foam exists in the bubble wall and the Plateau border. The heat transfer process in the gel foam layer is influenced by the gel foam microstructure. Poureslami et al. proposed a three-dimensional foam model to investigate the effects of geometrical parameters on heat transfer and flow characteristics, and established some correlations between the geometric parameter and flow losses and heat transfer rates [5]. Under the thermal radiation environment, the heat flux transmission in the gel foam layer mainly includes heat conduction and radiation. Heat conduction occurs in the gas and liquid phases [6,7]. Radiation thermal resistance mainly comes from the Plateau borders and the particles in the foam [3,8,9]. Heat convection in the gas phase can be neglected as the Rayleigh number related to the bubble size is far less than the critical value [10,11].

The main factors that affect the heat transfer of the three-phase gel foam layer are liquid phase fraction [4,12], sunscreen particles [11,13,14], and liquid phase evaporation [3,15]. The liquid phase content can affect the thermal conductivity and radiation thermal resistance of the gel foam layer. The apparent thermal conductivity of the gel foam without particles is affected by the thermal conductivity of the gas–liquid phase, the structural coefficient of the Plateau border, the bubble wall in the basic unit structure of foam, and the proportion of Plateau border in the liquid phase [16]. A higher liquid phase fraction in the gel foam layer and larger proportion of Plateau borders provide more absorption and scattering of thermal radiation, resulting in a greater radiation of thermal resistance [12]. However, the liquid phase content of gel foam is not the higher the better in engineering applications, considering the thermal resistance of the gel foam and the foaming volume [12]. The sunscreen particles in the gel foam can promote the absorption and scattering of the foam to the thermal radiation spectrum, thereby enhancing the radiation resistance of the gel foam layer [11,13]. The contribution of particles to the thermal resistance of the gel foam layer is mainly due to three factors [14,17,18]: the first one is the thermoluminescent properties of granular materials, which means the ability of absorption and scattering to thermal radiation spectrum; the second is the effect of the particle concentration. Zhou et al. investigated the effect of particle concentration, and the results showed that the thermal insulation performance was enhanced by increasing the particle concentration [14]. The third is the influence of the particle size, namely, the smaller the particle size at the same concentration, the greater the contribution to the thermal resistance. The commonly used sunshade materials include carbon black, SiC, TiO_2_, alumina, and ZrO_2_ [19,20,21,22]. Although carbon black, SiC, TiO_2_, alumina, ZrO_2_, and other materials have a relatively high ability of absorption and scattering to thermal radiation, the particle density is large, and the specific surface area is not high enough. These materials are more widely used in solid aerogels due to the difficulty of forming a stable three-phase gel foam with a water-based gel. Moreover, the cost of TiO_2_, alumina, ZrO_2_, and other material particles is higher compared with that of silica particles [23]. Therefore, this paper mainly studied the thermal insulation properties of a three phase gel foam containing silica nanoparticles.

Under the thermal environment, vaporization of the liquid phase in the gel foam absorbs heat and strengthens the endothermic effect of the foam layer. The surface of the foam layer near the high temperature environment gradually fades with the breakage of bubbles in the foam [3,15]. In the thermal insulation tests of the foam layer, the testing points of temperature and heat flux can only be arranged in limited positions. Accordingly, the variation characteristics of temperature and heat flux with the heating time can only be observed in limited positions. Nevertheless, complete data of heat flux distribution during the heating process can be obtained through numerical simulation. Boyd [3] established a 1D static calculation model of the heat transfer in a gas–liquid two-phase foam layer in a thermal radiation environment, which considered the effect of evaporation and heat absorption of the liquid phase. The results showed that the energy balance in the foam layer was controlled by absorption and evaporation. Said [24] calculated the radiation heat transfer in nanofluids containing TiO_2_ nanoparticles (particle size less than 20 nm). Considering the absorption and scattering effect to thermal radiation by the particles, Said’s model showed that the thermal radiation attenuation coefficient was proportional to the particle concentration. Manetti et al. [25] developed a solid–liquid heat transfer porous foam model, and the results showed that the foam thermal conductivity was influenced by solid and liquid thermal conductivity, the latent heat of vaporization, and the saturation temperature of the liquid. A number of important factors should be taken into account when creating a heat transfer model of the foam layer containing particles due to the special microstructure and heat transfer characteristics in the foam.

The existing studies have paid the most attention to the investigation of the heat transfer properties of gel foams through experimental methods, but numerical simulation is still lacking. In the present study, a three-phase heat transfer model of the gel foam layer containing silica nanoparticles was established. The characteristics of the layer thickness, phase distribution, and temperature distribution of the gel foam layer under radiation heating were studied numerically. The computation fluid dynamics (CFD) simulation was employed to investigate the effects of gas volume fraction, heating temperature, silica nanoparticle concentration, and particle size on the thermal stability and insulation performance of the gel foam layer.

## 2. Numerical Model

### 2.1. Simulation Domain and Boundary Conditions

**Figure 1:**
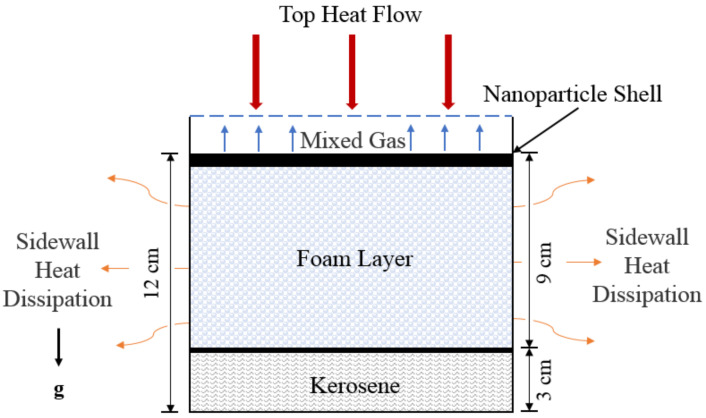
Simulation domain of the three-phase gel foam layer.

In Figure 1, the calculation domain of the model is divided into four subregions from top to bottom, which are the mixed gas layer, the shell layer of silica nanoparticles, the gel foam layer, and the kerosene layer. The liquid phase in the gel foam layer is mainly water, and the gas phase is air. The shell layer of the silica nanoparticles is formed during the collapse process of the gel foam layer.

### 2.2. Numerical Model of the Nanoparticle Shell

The thermal conduction process of the nanoparticle shell follows the thermal conduction differential equation, as shown in Equation (1)

(1) $$\rho_{sh}c_{sh}\frac{\partial T_{sh}}{\partial t}=\frac{\partial }{\partial x}\left(\lambda_{sh}\frac{\partial T_{sh}}{\partial x}\right)+\frac{\partial }{\partial y}\left(\lambda_{sh}\frac{\partial T_{sh}}{\partial y}\right)$$

where $\rho_{sh}$ is the density of the shell; $c_{sh}$ is the specific heat capacity of the shell; and $\lambda_{sh}$ is the thermal conductivity of the shell.

Silica nanoparticles accumulate to form the solid phase of the shell, while the gaps between particles filled by the mixed gas evaporating from the gel foam form the gas phase of the shell. The average thermophysical parameters of the shell can be calculated by the weighting method. The density and specific heat capacity of the shell are obtained by the weighted average of the density and the specific heat capacity of the gas and solid phases, respectively, as shown below

(2) $$\rho_{sh}=\varphi_{sh,s}\rho_{sh,s}+\varphi_{sh,g}\rho_{sh,g}$$

(3) $$c_{sh}=\frac{\varphi_{sh,s}\rho_{sh,s}c_{sh,s}+\varphi_{sh,g}\rho_{sh,g}c_{sh,g}}{\varphi_{sh,s}c_{sh,s}+\varphi_{sh,g}c_{sh,g}}$$

where $\varphi_{sh,s}\;\;\mathrm{and}\;\varphi_{sh,g}$ are the volume fractions of the solid and gas phases in the shell, respectively; $\rho_{sh,s}$ and $\rho_{sh,g}$ are the densities of the solid (i.e., silica) and gas phases in the shell, respectively. $c_{sh,s}$ is the specific heat capacity of the particles calculated by the Debye model [25]; as shown in Equation (5), $c_{sh,g}$ is the specific heat capacity of gas in shell, $c_{\mathrm{sh},g}=c_{mg}$, where $c_{mg}$ is the specific heat capacity of the mixed gas.

(4) $$c_{sh,s}=9\frac{R}{M}{\left(\frac{T_{sh}}{T_{D}}\right)}^{3}\int_{0}^{\frac{T_{D}}{T_{sh}}}\frac{\xi^{4}e^{\xi }}{{\left(e^{\xi }-1\right)}^{2}}d\xi$$

where *R* is the general gas constant; *M* is the molar mass of silica (M=60 g·mol^−1^); $T_{D}$ is the Debye temperature of silica [26,27]; ξ is an integral intermediate variable; and $T_{sh}$ is the temperature of the shell.

Assuming that there is no coupling between heat conduction and radiation in the inner voids of the shell, the radiation heat transfer is equivalent to the heat conduction process, and the overall thermal conductivity of the shell is obtained by

(5) $$\lambda_{sh}=\varphi_{sh,s}\lambda_{sh,s}+\varphi_{sh,g}\lambda_{sh,g}+\lambda_{r}$$

where $\lambda_{sh,s}$ is the thermal conductivity of shell solid phase; $\lambda_{sh,g}$ is the thermal conductivity of the shell gas phase; and $\lambda_{r}$ is the radiation equivalent thermal conductivity. The thermal conductivity of the gas mixture in the shell gap can be calculated by

(6) $$\lambda_{sh,g}=\frac{\lambda_{mg}}{1+\frac{2l_{mg}}{d_{sh,g}}}$$

where $\lambda_{mg}$ is the thermal conductivity of the gas mixture; $l_{mg}$ is the average free path of the mixed gas molecules; and $d_{sh,g}$ is the characteristic size of the pores in the shell. $d_{\mathrm{sh},g}=\frac{d_{p}-a}{2}$, where $d_{p}$ is the particle size and a is the characteristic size of the contact area between adjacent particles. The calculation of radiation equivalent thermal conductivity is shown as

(7) $$\lambda_{r}=4\varepsilon_{0}\sigma T_{\mathrm{sh}}{}^{3}\beta \delta_{\mathrm{sh},g}$$

where $\varepsilon_{0}=0.9$ is the surface emissivity of silica nanoparticles; σ is the Stefan–Boltzmann constant; $T_{\mathrm{sh}}$ is the temperature of the shell; β is the shape factor of the pore (*β* = 0.5); and $\delta_{sh,g}$ is the maximum width of the pore in the direction of heat transfer, $\delta_{sh,g\;}$=$\;2d_{\mathrm{sh},g}$.

As the silica nanoparticles are much smaller in size than the bulk silicon dioxide, their thermal conductivity is different. According to the kinetic theory formula, the expression for the thermal conductivity of nanoparticle shell is obtained by

(8) $$\lambda_{\mathrm{sh},s}=\frac{1}{3}\rho_{\mathrm{sh},s}c_{\mathrm{sh},s}v_{a}\Lambda$$

where $\rho_{sh,s}c_{sh,s}$ is the volume specific heat capacity of the nanoparticle shell; $v_{a}$ is the phonon group velocity (ν_a_ = 4100 m/s) [28]; and Λ is the mean free distance of the phonon.

**Figure 2:**
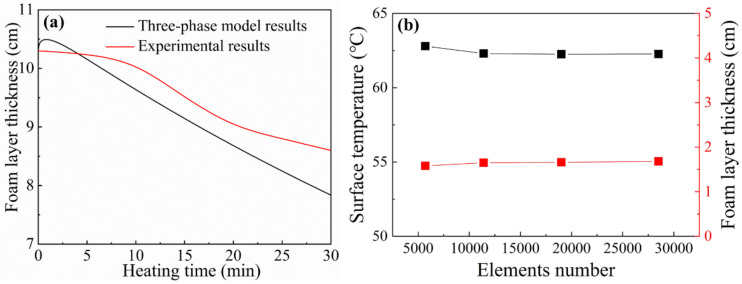
(**a**) Comparison between the numerical model and the experimental results [35] and (**b**) mesh independence verification.

The heat transfer of nanoscale solid phonons is influenced by three scattering effects, namely, the scattering caused by the thermal resistance of the volume of phonons, the scattering caused by the boundary of the solid structure unit, and the scattering caused by the interface of the solid structure unit. The mean phonon free path of the overall material is calculated according to Matthiessen’s Law [29,30], which is expressed as

(9) $$\frac{1}{\Lambda }=\frac{1}{\Lambda_{V}}+\frac{1}{\Lambda_{S}}+\frac{1}{\Lambda_{T}}$$

where $\Lambda_{V}$, $\Lambda_{S}$, and $\Lambda_{T}$ are the mean free paths of phonons caused by the above-mentioned three scattering processes, respectively.$\;\Lambda_{V}$ can be calculated by the thermal conductivity, the specific heat capacity, and the phonon group velocity of bulk silica [11], as shown in Equation (10)

(10) $$\Lambda_{V}=\frac{3\lambda_{\mathrm{bulk}\;}}{\rho_{\mathrm{bulk}\;}c_{\mathrm{bulk}}v_{a}}$$

where $\lambda_{bulk}$, $\rho_{bulk}$, and $c_{bulk}$ represent the thermal conductivity, density, and mass specific heat capacity of bulk silica, respectively. $\lambda_{bulk}=1.4$ W·m^−1^·K^−1^, and $c_{bulk}=920$ J·kg^−1^·K^−1^. According to Han [31], the average free path of the scattered phonons in the interface is calculated by

(11) $$\Lambda_{T}=3LT_{d}$$

where L (Figure 2) is the characteristic length of the structural unit of the particle group calculated by Equation (13); $T_{d}$ is the transmissivity of the phonon interface, $T_{d}=0.5$. The correlation between the diameter of the contact surface of adjacent particles and the characteristic length *L* of the structural unit of the particle layer can be analyzed by Equations (12) and (13) by referring to the Hertz contact formula in elastic mechanics.

(12) $${\left(\frac{a}{d_{p}}\right)}^{3}=\frac{3}{4}\pi \frac{1-\mu_{p}^{2}}{E_{p}}P_{0}{\left(1-\varphi_{\mathrm{sh},g}\right)}^{\frac{1}{x}}$$

(13) $$L=\sqrt{d_{p}^{2}-a^{2}}$$

where $d_{p}$ is the size of the nanoparticles; $\mu_{p}$ is the Poisson ratio of the silicon dioxide ($\mu_{p}=0.16$); $E_{p}$ is the elastic modulus of silica ($E_{p}=63\;$GPa); $P_{0}$ is the external pressure load ($P_{0}=1$ atm); x is an empirical constant (x=0.6); and $\varphi_{\mathrm{sh},g}\;$is the volume fraction of the gas phase in the shell ($\varphi_{\mathrm{sh},g}=0.9$). The calculation of the thermal conductivity of the aerogel solid skeleton was used for the phonon scattering caused by the boundary of solid structural units, assuming that the direction of heat flux is perpendicular to the interface of solid phase units, as shown in Equation (14):

(14) $$\Lambda_{s}=\frac{1+p}{1-p}a$$

where p is the mirror ratio of the boundary; a is the diameter of the interface between particles. Assuming that the boundary of the structural unit of the particle group and the interface of the interaction are diffuse scattering interfaces, then p=0.

### 2.3. Numerical Model of Three-Phase Gel Foam Layer

(a) Heat conduction equation

The heat conduction process of the three-phase gel foam layer follows the heat conduction differential equation, which is shown as:

(15) $$\rho_{f}c_{f}\frac{\partial T_{f}}{\partial t}=\frac{\partial }{\partial x}\left(\lambda_{f}\frac{\partial T_{f}}{\partial x}\right)+\frac{\partial }{\partial y}\left(\lambda_{f}\frac{\partial T_{f}}{\partial y}\right)+Q_{r}$$

where $\rho_{f}$ is the density of the gel foam layer; $c_{f}$ is the specific heat capacity of the gel foam layer; $\lambda_{f}$ is the equivalent thermal conductivity of the foam layer; and $Q_{r}$ is the radiation heat source item.

We assumed that the volume of the liquid, gas, and solid phases in the gel foam layer at the initial conditions were $V_{l}^{\text{’}}$, $V_{g}^{\text{’}}$, and $V_{s}^{\text{’}}$, respectively. Similar to the two-phase foam, the initial gas volume fraction $\varphi_{g}^{’}$, liquid volume fraction $\varphi_{l}^{\text{’}}$, and particle volume fraction $\varphi_{s}^{\text{’}}$ in the three-phase foam can be presented as follows, which numerically correspond to the initial foam expansion ratio E.

(16) $$\varphi_{g}^{\prime }=\varphi_{g|T_{f}=T_{0}}=\frac{V_{g}^{\prime }}{V_{l}^{\text{¢}}+V_{g}^{\prime }+V_{s}^{\text{¢}}}=1-\frac{1}{E^{\prime }}$$

(17) $$\varphi_{l}^{’}=\varphi_{l|T_{f}=T_{0}}=\frac{V_{l}^{’}}{V_{l}^{’}+V_{g}^{\prime }+V_{s}^{\prime }}$$

(18) $$\varphi_{s}^{\prime }=\varphi_{s|T_{f}=T_{0}}=\frac{V_{s}^{\prime }}{V_{l}^{’}+V_{g}^{\prime }+V_{s}^{\prime }}$$

where $\varphi_{g}$, $\varphi_{l}$, and $\varphi_{s\;}$ represent the volume fractions of the gas, liquid, and solid phases of the gel foam layer in the real-time state of the heating process, respectively. $T_{f}$ is the real-time temperature of the gel foam layer and $T_{0}$ is the ambient temperature, $T_{0}=293$ K. If the gas phase in the gel foam layer isobarically expands with the increasing temperature, while the thermal expansion of the liquid phase and solid phase is negligible, then:

(19) $$V_{g}=V_{g}^{\prime }\frac{T_{f}}{T_{0}}$$

(20) $$V_{l}=V_{l}^{\prime }$$

(21) $$V_{s}=V_{s}^{’}$$

where $V_{g}$, $V_{l}$, and $V_{s}$ are the volume variables of the gas, liquid, and solid phases in the gel foam layer in real-time state, respectively. Thus, the volume fraction of each phase can be deduced, which is related to the temperature and the initial volume of each phase, as shown in Equations (22)–(24)

(22) $$\varphi_{g}=\frac{V_{g}}{V_{f}}=\frac{V_{g}}{V_{l}+V_{g}+V_{s}}=\frac{V_{g}^{’}\frac{T_{f}}{T_{0}}}{V_{l}^{’}+V_{s}^{\prime }+V_{g}^{\prime }\frac{T_{f}f}{T_{0}}}$$

(23) $$\varphi_{l}=\frac{V_{l}}{V_{f}}=\frac{V_{l}}{V_{l}+V_{g}+V_{s}}=\frac{V_{l}^{\prime }}{V_{l}^{\prime }+V_{s}^{\prime }+V_{g}^{\prime }\frac{T_{f}}{T_{0}}}$$

(24) $$\varphi_{s}=\frac{V_{s}}{V_{f}}=\frac{V_{s}}{V_{l}+V_{g}+V_{s}}=\frac{V_{s}^{\prime }}{V_{l}^{\prime }+V_{s}^{\prime }+V_{g}^{\prime }\frac{T_{f}}{T_{0}}}$$

Then, $V_{f}=V_{l}+V_{g}+V_{s}$. The expressions of the real-time phase volume fraction with respect to the initial volume fraction and temperature of each phase are obtained by substituting Equations (16), (17), and (18) into Equations (22), (23), and (24), respectively. The result is shown as:

(25) $$\varphi_{g}=\frac{V_{g}}{V_{f}}=\frac{V_{g}}{V_{l}+V_{g}+V_{s}}=\frac{\varphi_{g^{’}}^{\prime }\frac{T_{f}}{T_{0}}}{\varphi_{g}^{\prime }\left(\frac{T}{T_{0}}-1\right)+1}$$

(26) $$\varphi_{l}=\frac{V_{l}}{V_{f}}=\frac{V_{l}}{V_{l}+V_{g}+V_{s}}=\frac{\varphi_{l}^{\prime }}{\varphi_{g}^{\prime }\left(\frac{T_{f}}{T_{0}}-1\right)+1}$$

(27) $$\varphi_{s}=\frac{V_{s}}{V_{f}}=\frac{V_{s}}{V_{l}+V_{g}+V_{s}}=\frac{\varphi_{s}^{\prime }}{\varphi_{g}^{\prime }\left(\frac{T_{f}}{T_{0}}-1\right)+1}$$

If the volume fractions of the gas phase and particles in the initial state are given, then the volume fraction of each phase of the gel foam at different temperatures and the various thermophysical parameters of the gel foam layer can be determined.

The density of the gel foam layer $\rho_{f}$ is given by the following equation:

(28) $$\rho_{f}=\frac{m_{f}}{V_{f}}=\frac{\rho_{l}^{’}\varphi_{l}^{\prime }+\rho_{g}^{\prime }\varphi_{g}^{\prime }+\rho_{s}^{\prime }\varphi_{s}^{\prime }}{\varphi_{g}^{\prime }\left(\frac{T_{f}}{T_{0}}-1\right)+1}$$

The total mass of the gel foam $m_{f}$ remains unchanged when the foam expands. $V_{f}$ is the volume of the gel foam layer in the real-time state. $\rho_{l}^{\text{’}}$, $\rho_{g}^{\text{’}}$, and$\;\rho_{s}^{\text{’}}$ are the densities of the liquid, gas, and particles in the initial state, respectively.

The specific heat capacity of the real-time gel foam layer $c_{f}$ is given by the following equation:

(29) $$c_{f}=c_{l}\theta_{l}+c_{g}\theta_{g}+c_{s}\theta_{s}$$

(30) $$\theta_{g}=\frac{\rho_{g}^{\prime }\varphi_{g}^{\prime }}{\rho_{l}^{\prime }\varphi_{l}^{\prime }+\rho_{g}^{\prime }\varphi_{g}^{\prime }+\rho_{s}^{\prime }\varphi_{s}^{\prime }}$$

(31) $$\theta_{l}=\frac{\rho_{l}^{\prime }\varphi_{l}^{\prime }}{\rho_{l}^{\prime }\varphi_{l}^{\prime }+\rho_{g}^{\prime }\varphi_{g}^{\prime }+\rho_{s}^{\prime }\varphi_{s}^{\prime }}$$

(32) $$\theta_{s}=\frac{\rho_{s}^{\prime }\varphi_{s}^{\prime }}{\rho_{l}^{\prime }\varphi_{l}^{\prime }+\rho_{g}^{\prime }\varphi_{g}^{\prime }+\rho_{s}^{\prime }\varphi_{s}^{\prime }}$$

where $c_{g}$, $c_{l}$, and $c_{s}$ are the specific heat capacity of the gas, liquid, and particles in the three-phase gel foam layer, respectively. $\theta_{g}$, $\theta_{l}$, and $\theta_{s}$ are the mass fractions of the gas, liquid, and particles in the gel foam layer, respectively.

The expression of the apparent thermal conductivity of the gas–liquid two-phase gel foam layer $\lambda_{\mathrm{gl}}$ is obtained according to the modified Torpey–Glicksman formula [16], as shown in Equations (33)–(36)

(33) $$\lambda_{\mathrm{gl}}=\frac{\lambda_{g}\frac{\varphi_{g}}{\varphi_{g}+\varphi_{l}}+\gamma \lambda_{l}\frac{\varphi_{l}}{\varphi_{g}+\varphi_{l}}}{\frac{\varphi_{g}}{\varphi_{g}+\varphi_{l}}+\gamma \frac{\varphi_{l}}{\varphi_{g}+\varphi_{l}}}$$

(34) $$\gamma =\left(1-f_{\mathrm{strut}\;}\right)\gamma_{\mathrm{wind}\;}+f_{\mathrm{strut}\;}\gamma_{\mathrm{strut}\;}$$

(35) $$\gamma_{\mathrm{strut}\;}=\frac{1}{3}\left(1+\frac{4\lambda_{g}}{\lambda_{g}+\lambda_{l}}\right)$$

(36) $$\gamma_{\mathrm{wind}\;}=\frac{2}{3}\left(1+\frac{\lambda_{g}}{2\lambda_{l}}\right)$$

where $\lambda_{\mathrm{gl}}$ is the apparent thermal conductivity of the gas–liquid two-phase foam; $\lambda_{g}$ and $\lambda_{l}$ are the thermal conductivities of the gas and liquid phases, respectively. $\lambda_{g}$ = 0.0259 W·m^−1^·K^−1^ and $\lambda_{l}\;$ = 0.5749 W·m^−1^·K^−1^. $\varphi_{g}$ and $\varphi_{l}$ are the volume fractions of the gas and liquid phases in the cell, respectively; $\gamma_{\mathrm{strut}}\;\mathrm{and}\;\gamma_{w\mathrm{ind}}$ are the structural coefficients of the Plateau border and bubble wall in the foam unit; γ is the volume average structural coefficients of the foam unit; and $f_{\mathrm{strut}}$ is the proportion of the Plateau border in the liquid phase of the foam, $f_{\mathrm{strut}}$ = 0.8.

Based on the correlation of the apparent thermal conductivity of gas–liquid two-phase gel foam, referring to the Maxwell model, and considering the influence of Brownian motion of nanoparticles on the thermal conductivity of the foam, the calculation formula of the apparent thermal conductivity of the three-phase gel foam [8,32] can be obtained, as shown in Equation (37)

(37) $$\lambda_{f}=\frac{\lambda_{s}+2\lambda_{\mathrm{gl}}-2\varphi_{s}\left(\lambda_{\mathrm{gl}}-\lambda_{s}\right)}{\lambda_{s}+2\lambda_{\mathrm{gl}}+\varphi_{s}\left(\lambda_{\mathrm{gl}}-\lambda_{s}\right)}\lambda_{\mathrm{gl}}+\frac{\rho_{s}\varphi_{s}c_{s}}{2}\sqrt{\frac{2k_{B}T}{3\pi d_{\mathrm{cp}}\mu_{\mathrm{gl}}}}$$

where $\lambda_{f}\;\mathrm{and}\;\lambda_{\mathrm{gl}}$ are the apparent thermal conductivities of the three-phase foam and gas–liquid two-phase foam, respectively; $\;\varphi_{s}$ is the volume fraction of the particles in the foam; $\rho_{s}=\rho_{\mathrm{bulk}}$ is the density of particles; $c_{s}=c_{\mathrm{sh},s}$ is the mass specific heat capacity of silica nanoparticles; $d_{\mathrm{cp}}$ is the particle size of the particle aggregate; $d_{\mathrm{cp}}=\left(2\sim 3\right)\times 10^{-7}$ m; $\mu_{\mathrm{gl}}$ is the average dynamic viscosity of the gas–liquid two-phase foam ($\mu_{\mathrm{gl}}=0.1\times 10^{-3}$ Pa·s); $k_{B}$ is the Boltzmann constant ($k_{B}=1.380649\times 10^{-23}$ J·K^−1^); and $\lambda_{s}$ is the thermal conductivity of silica nanoparticles suspended in the liquid phase given by:

(38) $$\lambda_{s}=\frac{\lambda_{\mathrm{bulk}}}{1+\frac{8\Lambda_{p}}{3d_{p}}}$$

where $\lambda_{\mathrm{bulk}}$ is the thermal conductivity of bulk silica; $\Lambda_{p}$ is the average phonon free path of silica nanoparticles; and $d_{p}$ is the size of the nanoparticle silica.

(b) Equation of radiation heat transfer

The heat radiation transfer process in the gel foam layer follows the classical heat radiation transfer equation [8,33]:

(39) $$\lambda_{s}=\frac{\lambda_{\mathrm{bulk}}}{1+\frac{8\Lambda_{p}}{3d_{p}}}\frac{dl(\overset{\text{→}}{r},\vec{s})}{ds}+\left(\kappa_{\mathrm{af}}+\kappa_{\mathrm{sf}}\right)I(\overset{\text{→}}{r},\overset{\text{→}}{s})=\kappa_{\mathrm{af}}n^{2}\frac{\sigma T_{f}{}^{4}}{\pi }+\frac{\kappa_{\mathrm{sf}}}{4\pi }\int_{0}^{4\pi }I\left(\overset{\text{→}}{r},{\overset{\text{→}}{s}}^{\prime }\right)\Phi \left(\overset{\text{→}}{s},{\overset{\text{→}}{s}}^{\prime }\right)d\;\Omega \text{ ′}$$

where I is the geothermal radiation intensity, which is related to the position vector $\overset{\text{→}}{r}$ and direction vector $\overset{\text{→}}{s}$. ${\overset{\text{→}}{s}}^{’}$ is the scattering direction vector, and s is the radiation path. $\kappa_{af}$ is the absorption coefficient, and $\kappa_{sf}$ is the scattering coefficient. *n* is the refractive index; σ is the Stefan–Boltzmann constant (i.e., blackbody radiation constant), $\sigma =5.672\times 10^{-8}$ W·m^−2^·K^−4^. $T_{f}$ is the local temperature of the foam layer, Φ is the phase function, which changes with the direction.  Ω ′ is the solid angle and $\left(\kappa_{a}+\kappa_{s}\right)s$ is the optical thickness of the medium.

The absorption coefficient of the foam layer to thermal radiation $\kappa_{\mathrm{af}}$ is related to the gas phase, liquid phase, and particles. The scattering coefficient $\kappa_{\mathrm{sf}}$ is related to the liquid phase and particle [17,18]. The absorption coefficient of the vapor phase (i.e., water vapor) $\kappa_{\mathrm{ag}}$ can be calculated by consulting the chart of water vapor emissivity and combining it with Bell’s law formula, taking $\kappa_{\mathrm{ag}}$ = 0.54 m^−1^. The absorption and scattering coefficients of the liquid phase in the model are calculated from the empirical correlations of the thermal radiation transfer of solid foam [33,34], which is related to the gas fraction, bubble diameter, and thermal radiation emissivity of the foam. The absorption and scattering coefficients of the particles can be calculated by referring to the empirical correlation of monodisperse particles [14,18]. The absorption and scattering coefficients of the three-phase foam for thermal radiation are shown as Equations (40) and (41), respectively.

(40) $$\kappa_{\mathrm{af}}=\frac{3\varepsilon_{r}\left(1-\varphi_{g}\right)}{2d_{\mathrm{pore}\;}}+\kappa_{\mathrm{ag}}\varphi_{g}+\frac{1.5\varphi_{p}Q_{a}}{d_{p}}$$

(41) $$\kappa_{\mathrm{sf}}=\frac{3\left(2-\varepsilon_{r}\right)\left(1-\varphi_{g}\right)}{2d_{\mathrm{pore}\;}}+\frac{1.5\varphi_{p}Q_{s}}{d_{p}}$$

where $\varepsilon_{\mathrm{rl}}$ is the thermal radiation emissivity of the liquid film and the Plateau border in the foam. Considering that the transmittance of the radiation of water on wavelengths below 1 μm is relatively high [3], we set $\varepsilon_{r}$ = 0.8 in the model. $\alpha_{g}$ is the porosity or gas phase fraction of foam. In the model, $d_{pore}$ is the diameter of the void or bubble, taking $d_{\mathrm{pore}}=0.001\;m$. $Q_{a}\;\mathrm{and}\;Q_{s}$ are the thermal radiation absorption and scattering efficiency factors of the particles, respectively, giving $\;Q_{s}$ = 0.27 [14]. $d_{p}$ is the particle size.

Therefore, the radiation source term in Equation (15) can be expressed as follows:

(42) $$Q_{r}=-\kappa_{a}\left[4\pi I_{b}-\int_{4\pi }I(s)d\Omega \right]$$

(c) Equation for the surface movement of the foam layer

During the collapse process of the foam layer, the upper surface of the foam layer continuously moves. According to the Stefan energy balance condition, the velocity of the upper surface of the foam layer $v_{c}$ resulting from the collapse of the foam layer is obtained.

(43) $$\rho_{\mathrm{ft}}\Delta Hv_{c}\times n=\left(q_{1}-q_{2}\right)\times n$$

where $\rho_{\mathrm{ft}}$ is the density of the upper surface of the foam layer; ΔH is the apparent phase transition latent heat at the upper surface of the foam layer; n is the normal vector on the upper surface of the foam layer;$\;q_{1}$ is the heat flux into the upper surface of the foam layer, and $q_{2}$ is the heat flux out of the upper surface of the foam layer. Hence, $q_{1}-q_{2}$ is the net heat flux into the upper surface of the foam layer.

The latent heat of the phase transition of the gel foam layer is different from that of pure water because of the large amount of gas in the gel foam layer. Thus, the latent heat of phase transition of the foam layer can be obtained by multiplying the mass fraction of water in the gel foam layer.

(44) $$\Delta H=\Delta H_{0}\theta_{l}$$

where ΔH is the apparent latent heat of phase transition of the foam layer; $\Delta H_{0}$ is the latent heat of phase change of pure water; and $\theta_{l}$ is the mass fraction of water in the gel foam layer.

The volume expansion of the foam layer after heating leads to additional velocity of the upper surface of the foam layer, namely, the expansion rate of the foam layer.

(45) $$v_{\mathrm{ex}}=\frac{\frac{\partial }{\partial t}\iint_{f}\varphi_{g}\alpha \left(T-T_{0}\right)dxdy}{\int_{l}ds}$$

where $v_{\mathrm{ex}}$ is the velocity of the upper surface of the foam layer caused by expansion; corner mark f represents the foam layer domain; α is the thermal expansion coefficient of ideal gas (α=1/273 1/K); and $\int_{l}ds$ is the total length of the phase interface.

The overall velocity of the upper surface of the gel foam layer is obtained by combining $v_{\mathrm{ex}}$ and $v_{c}$:

(46) $$v_{f}=v_{\mathrm{ex}}+v_{c}$$

The whole shell moves downward as the gel foam layer collapses. The shell gradually thickens due to the continuous precipitation of nanoparticles in the foam. Therefore, the moving velocities of the two surfaces on the top and bottom of the shell can be calculated

(47) $$v_{\mathrm{sh},\mathrm{up}}=v_{f}+v_{\mathrm{gr}}$$

(48) $$\left|v_{\mathrm{gr}}\right|=\frac{d}{dy}\left[\varphi_{g}\left(H_{0}-H\right)\right]$$

(49) $$v_{\mathrm{gr}}\cdot n^{\prime }=0$$

(50) $$v_{\mathrm{sh},\mathrm{lo}}=v_{f}$$

where $v_{\mathrm{sh},\mathrm{up}}$ and $v_{\mathrm{sh},\mathrm{lo}}$ are the moving velocities of the upper and lower surfaces of the shell, respectively. $v_{\mathrm{gr}}$ is the additional moving velocity on the upper surface of the shell resulting from the continuous collapse of the gel foam layer and the continuous precipitation of nanoparticles; $H_{0}$ and H are the initial and real thicknesses of the gel foam layer, respectively; and $n^{\prime }$ is the unit vector.

### 2.4. Numerical Model of the Kerosene Layer

The temperature difference in the kerosene layer is small because the volume heat capacity of the kerosene is large, and the heat flux through the foam layer is relatively small. Meanwhile, the Rayleigh number is also small. Accordingly, only the influence of heat conduction can be considered to calculate the heat transfer progress:

(51) $$\rho_{\mathrm{kero}\;}c_{\mathrm{kero}\;}\frac{\partial T_{\mathrm{kero}\;}}{\partial \tau }=\frac{\partial }{\partial x}\left(\lambda_{\mathrm{kero}\;}\frac{\partial T_{\mathrm{kero}\;}}{\partial x}\right)+\frac{\partial }{\partial y}\left(\lambda_{\mathrm{kero}\;}\frac{\partial T_{\mathrm{kero}\;}}{\partial y}\right)$$

where $\rho_{\mathrm{kero}}$, $c_{\mathrm{kero}}$, and $\lambda_{\mathrm{kero}}$ are the density, mass specific heat capacity, and thermal conductivity of the kerosene, respectively.

### 2.5. Numerical Model of the Mixed Gas Layer

The mass equation, momentum equation, and energy equation of the mixed gas flow are shown in Equations (52)–(55), respectively.

(52) $$\frac{\partial u}{\partial x}+\frac{\partial v}{\partial y}=0$$

(53) $$\begin{array}{c} \rho_{\mathrm{mg}}\left(\frac{\partial u}{\partial \tau }+u\frac{\partial u}{\partial x}+v\frac{\partial u}{\partial y}\right)=f_{x}-\frac{\partial P}{\partial x}+\mu_{\mathrm{mg}}\left(\frac{\partial^{2}u}{\partial x^{2}}+\frac{\partial^{2}u}{\partial y^{2}}\right) \end{array}$$

(54) $$\begin{array}{c} \rho_{\mathrm{mg}}\left(\frac{\partial v}{\partial \tau }+u\frac{\partial v}{\partial x}+v\frac{\partial v}{\partial y}\right)=f_{y}-\frac{\partial P}{\partial y}+\mu_{\mathrm{mg}}\left(\frac{\partial^{2}v}{\partial x^{2}}+\frac{\partial^{2}v}{\partial y^{2}}\right) \end{array}$$

(55) $$\begin{array}{c} \frac{\partial T_{\mathrm{mg}}}{\partial \tau }+u\frac{\partial T_{\mathrm{mg}}}{\partial x}+v\frac{\partial T_{\mathrm{mg}}}{\partial y}=\frac{\lambda_{\mathrm{mg}}}{\rho_{\mathrm{mg}}c_{\mathrm{mg}}}\left(\frac{\partial^{2}T_{\mathrm{mg}}}{\partial x^{2}}+\frac{\partial^{2}T_{\mathrm{mg}}}{\partial y^{2}}\right) \end{array}$$

where $\rho_{\mathrm{mg}}$, $c_{\mathrm{mg}}$, $\lambda_{\mathrm{mg}}$, and$\;\mu_{\mathrm{mg}}\;$are the density, specific heat capacity, thermal conductivity, and dynamic viscosity of the mixed gas, respectively. u and v are the speed of the x direction and y direction, respectively, and $T_{\mathrm{mg}}$ is the temperature of the mixed gas.

### 2.6. Model Validation

The model construction and calculation were based on the porous media heat transfer module in COMSOL 5.4. Considering that the computational domain of the model is two dimensional, the mesh can be drawn in enough detail without affecting the computational efficiency. The mesh independence was not an important part of the simulation in this work.

The experimental data from the previous study [35] were selected for comparison to validate the present model. In Tang’s experiment, the initial thickness of the three-phase gel foam layer was 10.3 cm. The initial volume fractions of the gas and particle phases were 0.87 and 0.026, respectively. The size of the nanoparticles was 15 nm, and the top heating temperature was 380 °C. Hence, the relevant three-phase gel foam heat transfer model parameters were set to be the same as the experimental data. In Figure 2a, the experimental results and the present model were in good agreement, which validates the present model. The difference may be caused by the difference in the top heating temperature between the numerical model and the experiment. Moreover, to verify the independence of the meshes, different numbers of elements were selected to calculate the cases at a heating temperature of 500 °C and the results are presented in Figure 2b. The surface temperature and foam layer thickness were evaluated after 10 min of heating. It was found that the surface temperature and foam layer thickness hardly changed with increasing grid number when the elements number reached above 10,000. The final element number used for our calculations was 11,400.

## 3. Results and Discussion

### 3.1. Characteristics of Foam Thickness and Heat Flux Distribution in the Gel Foam Layer

#### 3.1.1. Parameters from Typical Conditions

The typical heat transfer conditions of the gel foam layer exposed to thermal radiation and the operating parameters are shown in Table 1.

#### 3.1.2. Thickness Change of the Foam Layer

**Figure 3:**
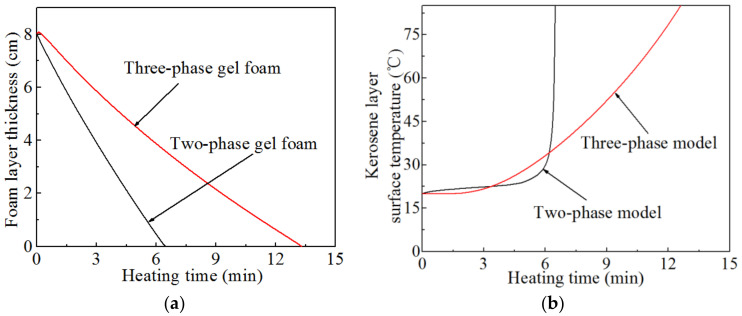
Comparison of the thermal stability and insulation of the two-phase and three-phase gel foam layer under the typical heating conditions. (**a**) Thickness change in the gel foam layer. (**b**) Surface temperature change in the kerosene layer.

To demonstrate the thermal stability of the gel foams, the variation curves of the thickness of the three-phase gel foam layer and the surface temperature of the kerosene layer with the heating time at the typical operating condition were calculated (Figure 3). The temperature of the liquid phase in the gel foam increased as the liquid phase absorbed thermal radiation. Then, the liquid phase rapidly evaporated after reaching the vaporization temperature. Accordingly, the interface of the three-phase gel foam gradually dissipated with the heating time. Specifically, the gel foam layer gradually subsided. In Figure 3a, the extinction velocity of the thickness of the three-phase foam layer was slower than that of the two-phase foam, indicating that the particles in the three-phase foam are helpful in enhancing the thermal stability and thermal resistance of the foam. The heat flux penetrates into the gel foam layer to the lower kerosene layer, resulting in an increase in the temperature of the kerosene layer (Figure 3b). The temperature rising rate of the kerosene layer below the three-phase foam layer was lower than that of the kerosene layer under the two-phase foam layer, indicating that the thermal insulation of the three-phase foam layer was better than that of the two-phase foam layer. Furthermore, Figure 3b shows that the thermal resistance of the two-phase foam layer rapidly decreased after the heat flux penetrating into the two-phase gel foam reached the kerosene layer, resulting in a sharp rise in the temperature of the kerosene layer. Hence, the insulation performance of the two-phase foam layer failed.

#### 3.1.3. Temperature in the Gel Foam Layer

**Figure 4:**
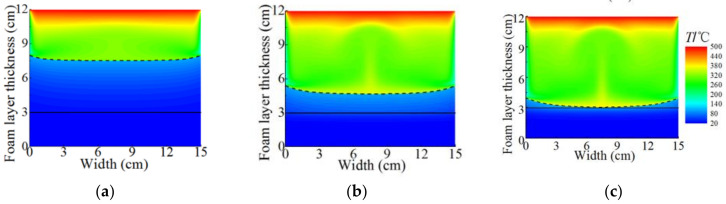
Temperature contour at different heating times under the typical heating conditions at different heating times. (**a**) *t* = 5 min, (**b**) *t* = 10 min, and (**c**) *t* = 13.31 min. The dotted line represents the upper surface of the gel foam layer.

The temperature distribution is a visual reflection of the insulation performance of the gel foam. To investigate the thermal insulation properties of the gel foam, the temperature contour of the foam layer at three different heating times were extracted, as shown in Figure 4. The three different heating time points was 5 min, 10 min, and 13.31 min, respectively. As shown in Figure 4, the dashed line represents the upper surface of the gel foam layer, which is always curved due to the presence of heat dissipation from the side walls. The solid line represents the upper surface of the kerosene layer. The upper surface of the foam layer moved downward with the heating time due to the evaporation of the liquid phase and the fracture of bubbles in the gel foam layer. The heat flux and temperature decreased with the increase in depth inside the foam layer due to the intrinsic endothermic effect of the upper foam layer. At the interface between the foam layer and the kerosene layer, the temperature remained nearly stable, about 20 °C, during the whole heating process. As the heating continued, the temperature in the foam layer increased constantly, as shown in Figure 3b. At the heating time of 13.31 min (Figure 4c), little gel foam remained on the kerosene layer when the foam layer temperature reached the maximum value.

#### 3.1.4. Volume Fraction of the Gas Phase

**Figure 5:**
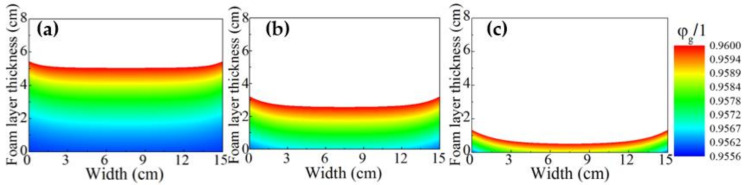
Contour of the gas phase volume fraction in the foam layer under the typical operating conditions at different heating times. (**a**) *t* = 4 min, (**b**) *t* = 8 min, and (**c**) *t* = 12 min.

The volume fraction of the gas phase contributes significantly to the thermal stability and insulation performance. To show the variation in the gas phase volume fraction with heating time, Figure 5 presents the contour of the gas fraction in the foam layer at three different heating times. The heating times were 4 min, 8 min, and 12 min, respectively. The radiation heat flux enters the foam layer from the top of the foam layer, resulting in a relatively high temperature in the upper part of the foam layer and a relatively low temperature in the lower part. The expansion degree of the gas phase in the upper part of the foam layer was larger than that in the lower part; hence, the gas content in the upper part was higher than that in the lower part. In Figure 5a, the gas content in the foam layer was divided into three intervals from top to bottom at different depths, which were 0.9575–0.9600, 0.9525–0.9575, and 0.9500–0.9525. At the same thickness, the gas content in the foam layer near the side wall was relatively small. Meanwhile, the gas content in the central area was relatively large. This condition was attributed to the model that had sidewall heat dissipation. The foam temperature near the side wall was lower than that in the central area, and the degree of thermal expansion of the gas phase was relatively small. When heated for 12 min, the residual foam layer had a higher gas volume fraction because the liquid phase evaporated, and the foam layer almost disappeared. Figure 5c also indicates that a large amount of heat flow passed through the foam layer, and the temperature in the foam layer was relatively high, so the insulation of the foam layer will fail.

#### 3.1.5. Thermal Diffusivity

**Figure 6:**
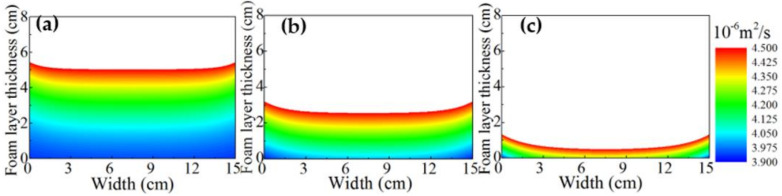
Contour of the foam layer thermal diffusivity under the typical operating conditions at different heating times. (**a**) *t* = 4 min, (**b**) *t* = 8 min, and (**c**) *t* = 12 min.

The thermal diffusivity of the conductive materials is the ratio of the thermal conductivity to the product of the density and specific heat capacity, which was employed to characterize the temperature homogenizing capability of the foam. To investigate the effect of heat time on the thermal diffusivity, the contour of the distribution of thermal diffusivity is shown in Figure 6. The heating times were 4 min, 8 min, and 12 min, respectively. As shown in Figure 6, the thermal diffusivity of the upper layer inside the foam was larger than that of the lower layer, showing obvious distribution inhomogeneity. The difference in the thermal diffusivity was caused by the inhomogeneity of the density distribution. Moreover, the thermal diffusivity also appeared to be inhomogeneous in the horizontal direction due to the presence of side wall heat dissipation. Similar to the distribution of the gas volume fraction as shown in Figure 5a, the distribution of thermal diffusivity in the foam layer could be roughly divided into two domains: the upper layer was in the range of $4.15\times 10^{-6}-4.5\times 10^{-6}$ m^2^ s^−1^, and the lower layer was in the range of $3.45\times 10^{-6}-4.15\times 10^{-6}$ m^2^ s^−1^, as shown Figure 6a. As the time increased, the temperature disturbance within the foam layer gradually transmitted downward, which was attributed to the continuous collapse of the foam layer.

### 3.2. Effect of Foam Composition and Operating Conditions

#### 3.2.1. Effect of the Top Heating Temperature

**Figure 7:**
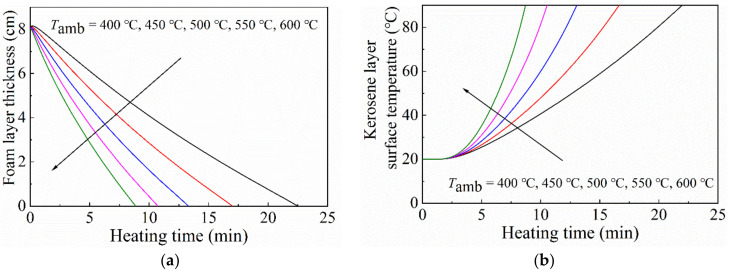
Curves of (**a**) the foam layer thickness and (**b**) surface temperature of the kerosene layer with different heating temperatures. The particle concentration was 1.0 vol%, and the particle size was 30 nm. The initial thickness of the foam layer was 8.0 cm.

In the model, the heating radiation intensity is controlled by the heating temperature at the upper boundary. According to the Stefan-Boltzmann law, the radiant heat flux exposed to the foam layer is proportional to the fourth power of the temperature. To investigate the effects of temperature on the thermal stability and insulation performance of the foam layer, the foam layer thickness and surface temperature of the kerosene layer are presented against the heating temperature in Figure 7. In the computational domain, the top heating temperature was set to 400 °C, 450 °C, 500 °C, 550 °C, and 600 °C , respectively. The particle concentration in the gel foam was 1.0 vol%, and the particle size was 30 nm. The initial thickness of the foam layer was 8.0 cm. When the heating temperature increased, the decreasing rate of the foam layer thickness increased, so the durability of the foam layer was reduced. As a result, the temperature rise rate of the kerosene layer surface increased significantly. Increasing the heating temperature could enhance the heat flux generating into the foam layer and weaken the stability of the foam. The above phenomenon is due to the fact that the heat flow, which is transferred by heat conduction, convection, and thermal radiation, increases with the increase in the heating temperature. In particular, the radiation heat flow increased proportionally to the fourth power of the top heating temperature.

#### 3.2.2. Effect of the Initial Gas Volume Fraction in Foam

**Figure 8:**
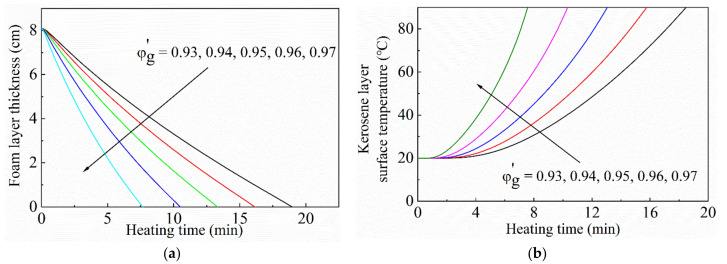
Curves of (**a**) the foam layer thickness and (**b**) surface temperature of the kerosene layer with different initial gas volume fractions. The particle concentration was 1.0 vol%, and the particle size was 30 nm. Initial thickness of the foam layer was 8.0 cm.

As the thermal conductivity of the gas phase is much lower than that of the liquid phase, the gas content in the foam has an important influence on the heat transfer or thermal insulation of the gel foam layer. To demonstrate the thermal stability and insulation performance of the foam layers with various initial gas volume fractions, the foam layer thickness and kerosene layer surface temperature with the initial gas contents of 0.93, 0.94, 0.95, 0.96, and 0.97 were calculated and are shown in Figure 8a,b, respectively. The particle concentration was 1.0 vol%, and the particle diameter was 30 nm. The initial thickness of the foam layer was 8 cm. As the initial gas content of the foam layer increased, the decreasing rate of the foam layer thickness increased, so the durability of the foam layer was reduced. Accordingly, the temperature rise rate of the kerosene layer surface increased significantly. At room temperature, increasing the initial gas content of the foam could strengthen the stability and durability of the foam. However, at high temperature, increasing the initial gas content will weaken the thermal stability and heat insulation performance of the gel foam layer.

There are three reasons to explain this phenomenon. First, the thermal diffusivity of the gas phase is much greater than that of the liquid phase. Accordingly, the apparent thermal diffusivity of the foam layer increases with the increase in the initial gas content. Second, the absorption and scattering coefficients of the liquid phase to thermal radiation are much larger than those of the gas phase. Therefore, the larger the initial gas content, the lower the liquid phase fraction, and the smaller the apparent thermal radiation resistance of the foam layer. Third, the increase in the initial gas content leads to the decrease in the latent heat per unit volume for vaporization of the foam layer.

#### 3.2.3. Effect of the Concentration of the Silica Nanoparticles

**Figure 9:**
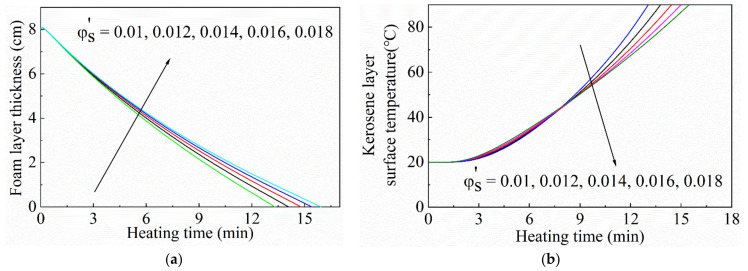
Curves of (**a**) the foam layer thickness and (**b**) surface temperature of the kerosene layer with different nanoparticle concentrations. The particle size was 30 nm. Initial thickness of the foam layer was 8.0 cm.

The silica nanoparticles in the gel foam could absorb and scatter the heat radiation. To investigate the effects of particle concentration on the thermal stability and insulation, the foam layer thickness and surface temperature of the kerosene layer were calculated. The particle concentrations were 1.0 vol%, 1.2 vol%, 1.4 vol%, 1.6 vol%, and 1.8 vol%, respectively. The particle size was 30 nm. The initial thickness of the foam layer was 8 cm. The initial gas phase volume fraction was 0.95. The durability and thermal insulation of the foam layer were characterized by the changes in the foam layer thickness and oil layer temperature, as shown in Figure 9. As the particle concentration of the foam layer increased, as presented in Figure 9a, the decreasing rate of the foam layer thickness decreased, so the durability of the foam layer increased. Accordingly, the temperature rise rate of the kerosene layer surface decreased. It was proven that the inside nanoparticles in foam play an important role in the stability and heat insulation performance of the foam layer. As shown in Figure 9b, in the initial stage of heating, the temperature rise rate of the kerosene layer surface decreased first with an increase in silica nanoparticles. After a period of heating, the temperature rise rate of the kerosene layer surface tended to slow down. This is because the addition of silica nanoparticles at the beginning of heating enhances the overall thermal diffusivity of the foam layer and strengthens the heat transfer performance of the foam layer. As the heating proceeds, there is a thin particle shell formed on the top surface of the foam layer with the foam collapsing in the heating progress, which is helpful to the thermal resistance of the foam layer.

#### 3.2.4. Effect of Size of the Silica Nanoparticles

**Figure 10:**
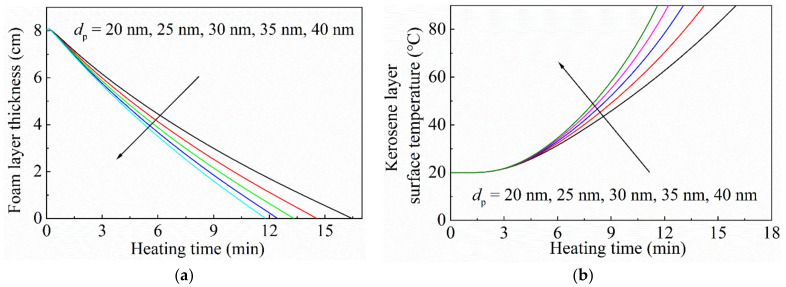
Curves of (**a**) the foam layer thickness and (**b**) surface temperature of the kerosene layer with different nanoparticle sizes. The particle concentration was 1.0 vol%. The initial thickness of the foam layer was 8.0 cm.

To investigate the effect of nanoparticle size on the durability and thermal insulation of the foam layer, the foam layer thickness and surface temperature of the kerosene layer are presented in Figure 10 as a function of heating time. The particle size was set at 20 nm, 25 nm, 30 nm, 35 nm, and 40 nm, respectively. The particle concentration was 1.0 vol%. The initial thickness of the foam layer was 8 cm. The initial gas phase volume fraction was 0.95. As the particle size in the foam increased, the decreasing rate of the foam layer thickness increased, so the durability of the foam layer decreased. Accordingly, the temperature rise rate of the kerosene layer surface increased. Increasing the size of the silica nanoparticles could weaken the absorbing and scattering effect of the particles on thermal radiation, so the thermal insulation performance of the foam layer decreased. Furthermore, small particles in the three-phase foam are favorable for the foaming efficiency and stability of the foam layer. Considering the foaming efficiency, stability, and heat insulation performance of the foam layer, small silica nanoparticles should be selected.

## 4. Conclusions

In this work, a heat transfer model of the gel foam layer with silica nanoparticles exposed to thermal radiation was established. The effects of the composition and structural characteristics of the foam on the heat transfer of the foam layer were studied. Through numerical simulation, the durability and heat insulation performance of the foam layer were theoretically analyzed. The influence of the top heating temperature, the initial gas content, the nanoparticle size, and concentration on the heat insulation performance of the gel foam layer were studied. The main conclusions are as follows:

- During the heating progress, the foam layer thickness decreases gradually with the increase in the kerosene layer surface temperature.
- On the premise of ensuring the foaming expansion ratio, decreasing the initial gas content could enhance the thermal stability and heat insulation performance of the gel foam layer.
- Small size and high concentration nanoparticles are preferred to the thermal stability and heat insulation performance of the three-phase gel foam.

## Figures and Tables

**Table 1:** Parameters of the typical operating conditions.

Parameter	Symbol/Unit	Set Value
Heating temperature on top	$T_{\mathrm{amb}}{/}^{{}^{\circ}}C$	500
Convective heat transfer Coefficient at the wall	$h_{f}/W\cdot m^{-2}\cdot K^{-1}$	10
Initial volume fraction of the gas phase in foam	$\varphi_{g}^{\prime }/1$	0.95
Initial volume fraction of the particle phase in foam	$\varphi_{s}^{\prime }/1$	0.01
Size of silica nanoparticles	$d_{p}/m$	3 × 10^−8^
Average diameter of bubbles in foam	$d_{\mathrm{pore}\;}/m$	1 × 10^−3^
Liquid volume fraction of the wall in the foam cell	$f_{w}/1$	0.8
Initial thickness of the foam layer	$H_{0}/\mathrm{cm}$	8

## Data Availability

Not applicable.

## Nomenclature

*c*	Specific heat capacity, J·kg^−1^·K^−1^
*d*	Size parameter
*E*	Foam expansion ratio
*f*	Volume fraction of particle
*H*	Thickness of the foam layer, cm
*m_f_*	Total mass of the gel foam layer, kg
*Q*	Efficiency factor
*q_r_*	Radiant heating flux, kW·m^−2^
*T*	Temperature, K
*V*	Volume, m^3^
*v*	Velocity, m·s^−1^
**Greek symbols**	
*γ*	Structure coefficient
*θ*	Mass fraction of the gas, liquid, or solid phase, %
*κ*	Scattering or absorption coefficient, m^−1^
Λ	Mean free path of phonon, m
*λ*	Thermal conductivity, W·m^−1^·K^−1^
*ρ*	Density, kg·m^−3^
τ	Heating time, s
φ	Volume fraction of gas, liquid, or solid phase
**Subscripts**	
*bulk*	Body phase
f	Foam layer
*g*	Gas phase
*l*	Liquid phase
*p*	Particle phase
*r*	Radiation
*s*	Solid phase
sh,lo	Lower surface of shell
sh,up	Upper surface of shell
strut	Plateau border of foam cell
wall	Wall of foam cell
